# *Pseudaspidimerus palatus*, a new species of the genus *Pseudaspidimerus* Kapur, 1948 from the Malay Peninsula (Coleoptera, Coccinellidae)

**DOI:** 10.3897/zookeys.706.18081

**Published:** 2017-10-04

**Authors:** Lizhi Huo, Wenjing Li, Xingmin Wang

**Affiliations:** 1 Key Laboratory of Bio-Pesticide Innovation and Application, Engineering Technology Research Center of Agricultural Pest Biocontrol, Guangdong Province; College of Agriculture, South China Agricultural University, Guangzhou 510640, China

**Keywords:** Aspidimerini, lady beetles, new record, Malaysia, Singapore

## Abstract

A new species of the genus *Pseudaspidimerus* Kapur, 1948 (Coleoptera: Coccinellidae), *Pseudaspidimerus palatus* Huo & Wang, **sp. n.** from the Malay Peninsula is described with illustrations and a distribution map. The genus *Pseudaspidimerus* is recorded for the first time from Malaysia and Singapore.

## Introduction

**Figure 1.**
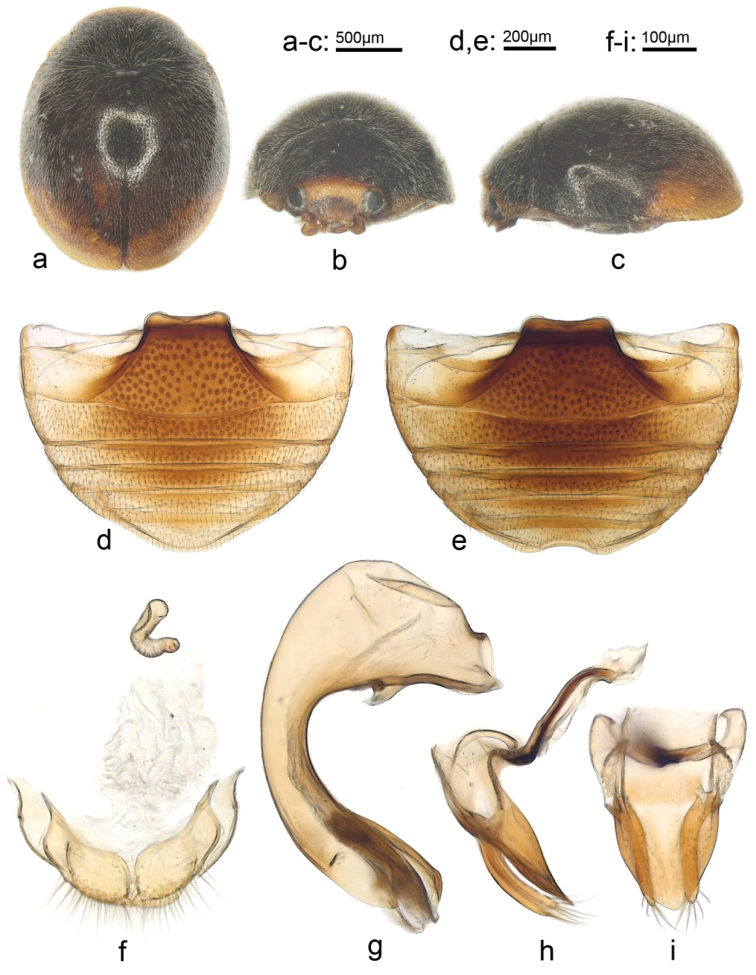
*Pseudaspidimerus palatus* Huo & Wang sp. n. **a** dorsal view **b** frontal view **c** lateral view **d** female abdomen **e** male abdomen **f** female genitalia **g** penis **h** lateral view of tegmen **i** ventral view of tegmen.

**Figure 2.**
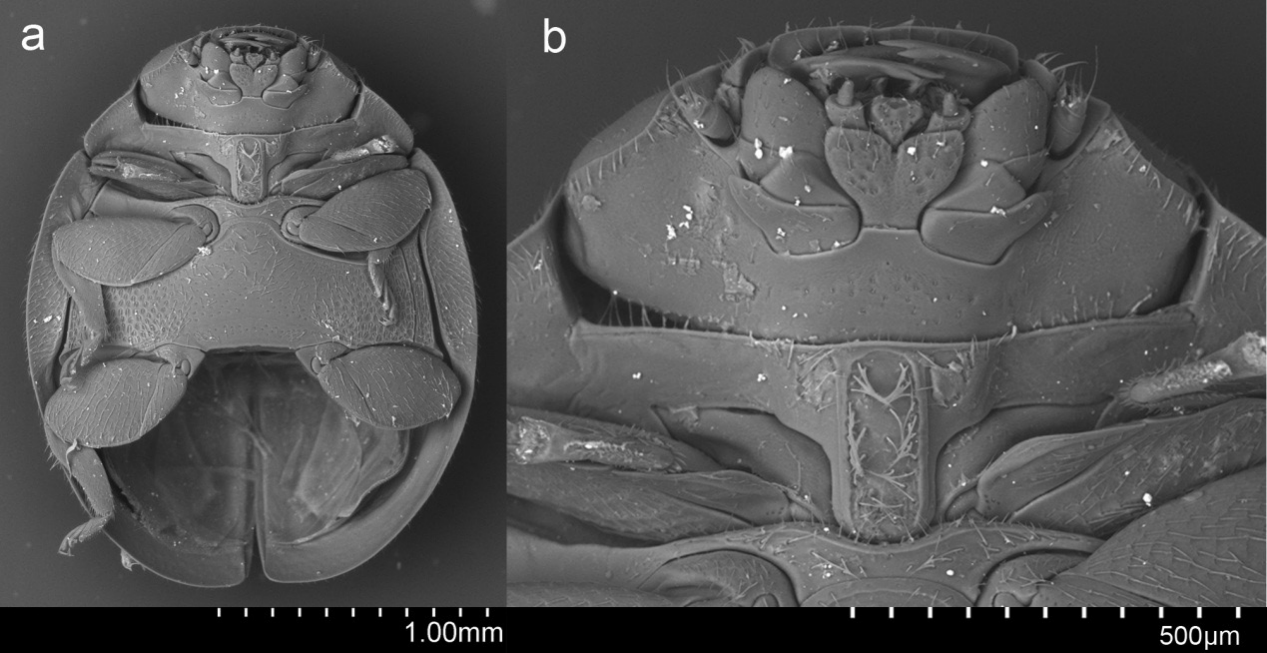
*Pseudaspidimerus palatus* Huo et Wang sp. n. **a** ventral view **b** prosternum and mouth part.

*Pseudaspidimerus* Kapur is a small genus of the tribe Aspidimerini Weise, 1900, with only ten species described until now (Poorani 2001; Huo et al. 2014). Species of this genus mostly prey on aphids, mealybugs, scale insects, and whiteflies, and are important natural enemies of economically important pests of various crops (Poorani 2001; Basu and Patro 2007; Megha et al. 2015). Kapur (1948) established this genus based on its parallel prosternal carinae forming a rectangular area (Fig. 2b) and its robust penis (Fig. 1g), which are the most important generic characters. During the course of our study of borrowed specimens from different repositories, a new species of this genus was discovered from the Malay Peninsula. This is the first record of the genus *Pseudaspidimerus* from Malaysia and Singapore.

## Materials and methods

All studied materials were borrowed from the following museums:

**HNHM** Hungarian Natural History Museum, Budapest, Hungary;

**MIZ** Museum and Institute of Zoology PAS, Warsaw, Poland;

**NMPC** Natural History Museum, Prague, Czech Republic.

Type specimens are deposited in the above museums, except two paratypes kept in South China Agricultural University (**SCAU**).

Dry specimens were softened in 70°C water for 12 hours; the abdomen was detached and cleared in warm 10% KOH solution for 1–2 hours. Genitalia of both sexes were dissected, rinsed with distilled water, transferred to glycerol, and examined on slides. Genitalia images were photographed with digital cameras (Axiocam 506 color) connected to the microscope (ZEISS Imager M2). The software ZEN 2.3 was used to capture genitalia images.

External morphology was observed with a stereomicroscope (SteREO Discovery V20, Zeiss). Images were photographed with digital cameras (AxioCam HRc) connected to the microscope. Measurements were made using the measurement tools of the software AxioVision Rel. 4.8. The following abbreviations are used in the description:

**TL** total length, from apical margin of clypeus to apex of elytra;

**TW** total width, across both elytra at widest part;

**TH** total height, through the highest point of elytra to metaventrite;

**HW** head width, including eyes;

**PL** pronotal length, from the middle of anterior margin to the base of pronotum;

**PW** pronotal width at widest part;

**EL** elytral length, along the suture, from the apex to the base including the scutellum;

**EW** elytral width, across both elytra at widest part;

**ID** interocular distance, nearest distance between eyes.

Scanning electron micrographs were made using HITACHI S-3400N in the Electron Microscopy Laboratory of the MIZ. The distribution map was downloaded from a free map website (http://alabamamaps.ua.edu) and all images were cleaned up and laid out in plates with Adobe Photoshop CS5. Morphological terms follow Ślipiński and Tomaszewska (2010).

## Taxonomy

### Pseudaspidimerus palatus

Taxon classificationAnimaliaColeopteraCoccinellidae

Huo & Wang sp. n.

http://zoobank.org/7961DD3D-2423-42E1-A7E5-35C53C0A4612

Figures 1 , 2 , 3

#### Types.

**Holotype**: 1♂, “Thailand, Ranong prov. Ban Na env., 22-26.III.1996, 9°34'N, 98°42'E, K Majer leg” (NMPC); **Paratypes** (5): 2♀, “S Thailand, Betong Gunung Cang dun vill., Yala dist., 25.3.-22.4.1993, J. Horák leg” (NMPC, SCAU); 2♂, “Singapore, 29 Dr. Baum” (MIZ, SCAU); 1♀, “Malacca Biró/ Kwala-Lumpur/” (HNHM).

#### Diagnosis.

Elytra black with apical part yellowish brown, maculae oblique expending from apical 1/3 of suture to lateral 1/2 length of lateral margin; Penis extremely robust, arcuate, swollen anteriorly with small inner branch, narrowest at middle; Penis guide, in lateral view, widest at base, gradually narrowing to pointed apex. In ventral view, shovel shaped, only a little longer than broad, slightly narrowing to basal 1/3, thence gradually narrowing to small rounded apex.

#### Description.

TL: 1.91–2.07 mm, EL: 1.43–1.49 mm, TW: 1.56–1.66 mm, TH: 1.00–1.06 mm, PL: 0.64–0.73 mm, PW: 1.13–1.25 mm, HW: 0.76–0.81 mm, ID: 0.40–0.43 mm, TL/TW: 1.22–1.25, PL/PW: 0.57–0.58, EL/EW: 0.90–0.92, HW/PW: 0.65–0.67, PW/EW: 0.72–0.75, ID/HW: 0.53.

***Body*** oblong oval, densely covered with short, silvery white pubescence (Fig. 1a). Head yellow in male (Fig. 1b) and black in female. Pronotum black with anterior margin narrowly reddish brown and ante-lateral corners with a small triangular yellow spot. Elytra black with apical part yellowish brown, maculae oblique expending from apical 1/3 of suture to lateral 1/2 length of lateral margin. Underside dark brown to black except mouthpart, prothoracic hypomeron, and legs yellow. Mentum dark brown. Posterior margin of ventrite 6 strongly emarginate in male and broadly rounded in female (Fig. 1d–e). Punctures on frons fine and dense, 0.5–1.0 diameters apart; on elytra and pronotum sparse, 1.0–2.0 diameters apart; on metaventrite fine and sparse on middle part, coarse and densely distributed laterally (Fig. 2a). Ventral surface with short, dense, silvery pubescence.

***Male genitalia.*** Penis extremely robust, arcuate, swollen anteriorly with small inner branch, narrowest at middle (Fig. 1g). Tegminal strut slightly shorter than main part of tegmen. Parameres slightly longer than phallobase length and a little shorter than penis guide, apex with long sparse setae (Fig. 1h). Penis guide, in lateral view, widest at base, gradually narrowing to pointed apex. In ventral view, shovel shaped, only a little longer than broad, slightly narrowing to basal 1/3, thence gradually narrowing to small rounded apex (Fig. 1i).

***Female genitalia.*** Coxites fairly broad, 0.5 times as long as wide, with a projection on basal end, apical and outer margin with dense, long setae. Spermatheca curved, C-shaped, ramus and nodulus not clearly differentiated (Fig. 1f).

#### Distribution.

Thailand (Ranong, Yala), Malaysia (Kuala Lumpur), Singapore.

#### Remarks.

In general appearance, this species is similar to a variation of *P. mauliki* (Huo et al. 2014, Fig. 2e) with elytral apical third with an oblique yellowish brown spot laterally extending up to nearly the half length of elytra. The penis of male genitalia is also very similar to that of *P. mauliki* (Huo et al. 2014, Fig. 2j): penis extremely robust, arcuate, swollen anteriorly with a small inner branch, narrowest at middle. The only difference is the ventral view of penis guide. In *P. mauliki*, penis guide is about 1.2 times as long as broad at base, widest at basal third and then gradually narrowing to a fairly broad apex, which is weakly emarginate at middle (Huo et al. 2014, Fig. 2k). In *P. palatus*, penis guide is shovel shaped, only a little longer than broad, widest at basal third, gradually narrowed thereafter to a rounded apex. The shape of the penis guide is also close to that of *P. lambai* Kapur, 1967 and *P. flaviceps* (Walker, 1859). In *P. lambai* and *P. flaviceps*, penis guide is more slender in lateral view and longer in ventral view, and about two times as long as broad.

#### Etymology.

The specific name is derived from the Latin noun “pala” and postfix “-atus”, referring to that its penis guide looks like a shovel in ventral view.

**Figure 3.**
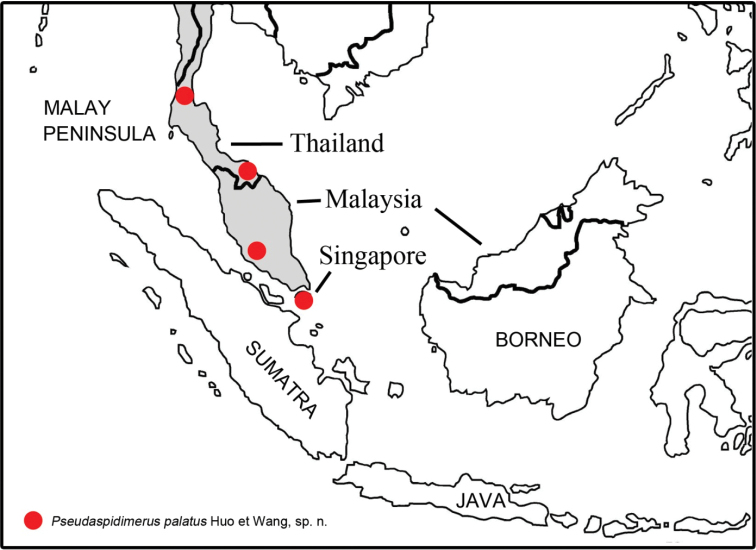
Distribution map of *Pseudaspidimerus palatus* Huo & Wang, sp. n.

## Supplementary Material

XML Treatment for Pseudaspidimerus palatus

## Supplementary materials

Supplementary material 1 Table of localities Data type: occurence This dataset is made available under the Open Database License (http://opendatacommons.org/licenses/odbl/1.0/). The Open Database License (ODbL) is a license agreement intended to allow users to freely share, modify, and use this Dataset while maintaining this same freedom for others, provided that the original source and author(s) are credited.Lizhi Huo, Wenjing Li, Xingmin Wang
